# Depressive Symptoms Profiles and Cognitive Outcomes After Stroke

**DOI:** 10.1002/brb3.70801

**Published:** 2025-09-15

**Authors:** Giuseppe Scopelliti, Francesco Mele, Ilaria Cova, Federico Masserini, Valentina Cucumo, Giorgia Maestri, Alessia Nicotra, Arianna Forgione, Pierluigi Bertora, Simone Pomati, Emilia Salvadori, Leonardo Pantoni

**Affiliations:** ^1^ Neurology Unit Luigi Sacco University Hospital Milan Italy; ^2^ Neuroscience Research Center Department of Biomedical and Clinical Sciences University of Milan Milan Italy; ^3^ Department of Neurorehabilitation Casa Di Cura Igea Milan Italy

**Keywords:** anxiety, apathy, depression, stroke, vascular cognitive impairment

## Abstract

**Introduction:**

Post‐stroke depressive symptoms are heterogeneous and variably associated with other psycho‐cognitive features. We employed cluster analysis to identify distinct profiles of post‐stroke depressive symptomatology and their association with cognitive performance.

**Methods:**

We included consecutive patients undergoing neuropsychiatric evaluation 6 months after stroke. Cluster analysis incorporated the Center for Epidemiologic Studies Depression Scale, along with the apathy and anxiety items from the Neuropsychiatric Inventory questionnaire. Baseline clinical/neuroimaging variables and 6‐months cognitive outcomes were compared across profiles.

**Results:**

We included 189 patients with acute cerebrovascular events (median age 75.4 years, 62% male, 80% ischemic strokes). Three profiles emerged: (A) low‐depressive symptoms (*n* = 108), (B) moderate‐depressive symptoms plus anxiety (*n* = 41), (C) high‐depressive symptoms plus apathy (*n* = 40). Regarding baseline predictors of 6‐month depressive symptoms profiles, patients with high‐depressive symptoms plus apathy exhibited lower Montreal Cognitive Assessment scores at baseline (16.0 vs. 21.5; adjusted odds ratio [adj.OR] per 1‐point increase 0.91, 95% confidence interval [95% CI] 0.83–0.99) compared to patients with low‐depressive symptoms; moderate‐depressive symptoms plus anxiety patients had less cortical atrophy compared to both low‐depressive symptoms (adj.OR 0.92, 95% CI 0.86–0.99) and high‐depressive symptoms plus apathy (adj.OR 0.89, 95% CI 0.81–0.97) profiles. Regarding 6‐month cognitive performance, high‐depressive symptoms plus apathy patients showed higher rates of post‐stroke dementia and attention/executive function impairment compared with the two other groups (both *p* < 0.05), and higher rates of language impairment compared with low‐depressive symptoms profile (*p *< 0.05).

**Conclusion:**

By integrating apathy and anxiety in our model, depressive symptoms after stroke emerged as heterogeneous neuropsychiatric syndromes, showing different baseline predictors and distinctive cognitive patterns.

## Graphical abstract

We included 189 consecutive patients undergoing neuropsychiatric screening six months after stroke. Cluster analysis incorporated the *z*‐scores of Center for Epidemiologic Studies Depression Scale (CES‐D) and apathy and anxiety item scores from the Neuropsychiatric Inventory questionnaire (NPI‐q). Three distinct profiles emerged:

These three neuropsychiatric profiles exhibited distinct cognitive profiles, suggesting how post‐stroke depressive symptoms represent heterogeneous neuropsychiatric syndromes.

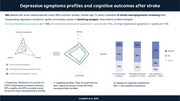

## 1 Introduction

Depressive symptoms are a frequent and debilitating consequence of stroke, significantly affecting recovery and quality of life (Towfighi et al. 2017; Kutlubaev and Hackett 2014). Despite extensive research, the etiology of post‐stroke depression remains complex and multifaceted, suggesting that depressive symptoms after stroke likely represent a spectrum of syndromes with distinct underlying substrates (Xiao et al. 2024; Ayerbe et al. 2013). This heterogeneity underscores the necessity for diagnostic and therapeutic approaches tailored to individual patient profiles.

Differentiating post‐stroke neuropsychiatric syndromes is crucial but often challenging, due to overlapping symptoms and shared pathophysiological mechanisms (Zhang et al. 2020). Depression and anxiety frequently co‐occur in stroke survivors, contributing to the overall burden of illness and complicating treatment strategies; while anxiety may stem from psychological responses to the stroke event and its aftermath, it also involves neurobiological changes that intersect with depressive pathways (Ayerbe et al. 2014; Scopelliti et al. 2022a). Apathy, despite the overlap with depressive manifestations, is less associated with mood disturbances and more with deficits in goal‐directed behavior, suggesting a different neural basis and a link with neurodegeneration and cognitive decline (Tay et al. 2021). While different neuropsychiatric syndromes may fall under a diagnosis of depression, recognizing the distinct features underlying post‐stroke depressive symptoms is relevant for improving patient management and outcomes, as neuroimaging and clinical data suggest that diverse neurobiological mechanisms may contribute to these conditions, showing different functional and cognitive correlates (Castello et al. 2022; Scopelliti et al. 2024).

We hypothesized that employing a cluster analysis that incorporates the evaluation of anxiety and apathy might elucidate the complexity of post‐stroke depressive symptoms and identify the distinct neuropsychiatric profiles arising after a cerebrovascular event. By exploring the differences in terms of cognitive outcomes, we seek to enhance the understanding of the multifactorial nature of post‐stroke depressive symptoms and pave the way for more targeted and effective diagnostic and therapeutic pathways.

## 2 Methods

### 2.1 Study Design and Patient Inclusion

We included consecutive patients hospitalized for an acute cerebrovascular event in the stroke unit of the Luigi Sacco Hospital in Milan, Italy, from January 2019 to December 2022 who underwent a standard neuropsychiatric evaluation 6 months after discharge. All patients admitted were screened for inclusion in our prospective registry. Enrollment was stopped between March and August 2020 and between November 2020 and October 2021 because of the COVID‐19 pandemic reorganization of the service. The study protocol and design were previously described (Cova et al. 2022; Cova et al. 2024; Mele et al. 2024).

### 2.2 Baseline Clinical and Radiological Data

Demographic and clinical variables, such as age, sex, education, and vascular risk factors were recorded. Type of the acute cerebrovascular event was categorized as ischemic stroke, transient ischemic attack (TIA), intracerebral hemorrhage, and other stroke type (the latter term encompassing cerebral venous thrombosis, subarachnoid hemorrhage, dural fistula, and transient focal neurological episodes). Severity of stroke symptoms was evaluated using the National Institutes of Health Stroke Scale (NIHSS) score. Pre‐stroke disability was defined as a Modified Rankin Scale (mRS) score > 2. Two trained neuropsychologists administered to informants the 16‐item Informant Questionnaire on Cognitive Decline in the Elderly (IQCODE) for the assessment of pre‐stroke cognitive status (Jorm 1994). Pre‐stroke depressive symptoms were assessed during the hospital stay by administering the Neuropsychiatric Inventory Questionnaire (NPI‐Q) to relatives or caregivers, with specific reference to the period preceding the index event (Cummings et al. 1994). We assessed cognitive status in the acute phase using the Montreal Cognitive Assessment (MoCA), with scores ranging 0–30 (Nasreddine et al. 2005). The MoCA scores were adjusted according to national normative data (Aiello et al. 2022).

Patients underwent brain imaging during hospitalization with either computed tomography (CT) or a 1.5 Tesla magnetic resonance imaging (MRI), as required by clinical practice. A trained and certified neurologist, who was blinded to the pre‐event clinical status assessments, evaluated the neuroimaging features. We reported cerebral atrophy using the Global Cortical Atrophy (GCA) scale on CT scans, with total scores ranging 0–39 (Pasquier et al. 1996; Hobden et al. 2024). We assessed white matter hyperintensities severity using van Swieten scale (total scores ranging 0–4) on CT scans, and Fazekas scale (total scores ranging 0–6) on MRI scans (Fazekas et al. 1987; Van Swieten et al. 1990).

We recorded the presence of cortical superficial siderosis (CSS) and presence of cerebral microbleeds (CMB) on brain MRI scans according to STRIVE criteria (Wardlaw et al. 2013).

### 2.3 Follow‐Up Evaluation

An extensive neuropsychological evaluation was carried out by trained neuropsychologists 6 months after the index event for all included patients (Cova et al. 2024). We used the Center for Epidemiologic Studies Depression (CES‐D) scale to evaluate the severity of depressive symptoms and the NPI‐Q to screen for apathy and anxiety. The CES‐D is a well‐recognized screening tool for detecting depressive symptoms in stroke patients (Shinar et al. 1986). The NPI‐Q is an informant‐based questionnaire consisting of 12 items (hallucinations, delusions, agitation, depression, anxiety, elation, apathy, disinhibition, irritability, aberrant motor behavior, nighttime behavior, and appetite changes), that provides a quick assessment of neuropsychiatric symptoms (Cummings et al. 1994). For each item, the informant reported whether the principal symptoms were absent or present, and if present, their severity (on a scale of 1–3). Patients who attended the 6‐month follow‐up visit but for whom neuropsychiatric screening could not be fully conducted—either because they were unsuitable for CES‐D assessment or there was no reliable informant for the NPI‐Q—were excluded from the study. A comprehensive neuropsychological battery was administered, and cognitive test results were evaluated using national normative studies that applied the equivalent score (ES) methodology (Capitani and Laiacona 1997). ES methodology is a nonparametric norming method based on percentiles distributions, and scores range from ES = 0 (impaired performance, i.e., adjusted score below the outer confidence limit for the fifth centile of the normal population), to ES = 1 (borderline performance, i.e., adjusted score between the outer and inner confidence limits for the fifth centile of the normal population), and ES = 2–4 (normal performance).

The impairment in a specific cognitive domain (memory, visuospatial ability, language, attention/executive function) was defined as an ES of 0 or 1 in at least one of the tests within that domain (see Table S1 for a description of the neuropsychological tests used). We determined the presence of post‐stroke cognitive impairment (PSCI) at the 6‐month follow‐up, classifying it as either mild cognitive impairment (MCI) or dementia, based on neuropsychological battery results and a neurologist's clinical judgment as detailed hereafter:

‐MCI was diagnosed when at least two tests showed an ES of 0 or 1 in patients who completed at least four tests covering at least three cognitive domains, without evidence of functional impairment in daily life activities.
‐Dementia was diagnosed either (i) when at least two tests showed an ES of 0 or 1 in patients who completed at least four tests covering at least three cognitive domains and demonstrated functional impairment in daily life activities, or (ii) when the inability to complete the tests was due to clinically evident cognitive impairments with a functional impact.

Post‐stroke functional status was assessed using mRS, and disability was defined as a score > 2.

### 2.4 Statistical Analysis

Quantitative variables were presented as medians with interquartile ranges (IQR), while categorical variables were expressed as frequencies (percentages). Baseline characteristics were compared between patients included and excluded from the analyses using Chi‐square test for categorical variables and Mann–Whitney *U* test for quantitative variables. Three neuropsychiatric scales were used to classify patients into homogenous subgroups based on depressive symptoms, apathy, and anxiety scores at 6 months: the NPI‐Q Apathy subscale, the NPI‐Q Anxiety subscale, and the CES‐D. To ensure comparability across these different scales, each score was normalized into *z*‐scores. This normalization involved calculating the mean and standard deviation for each scale and then transforming the raw scores into standardized scores. Following normalization, a k‐means cluster analysis was performed to identify distinct subgroups within the sample based on their neuropsychiatric profiles. For this study, we a priori selected a number of three clusters, based on findings from a previous study on post‐intracerebral hemorrhage depressive symptom profiles (Scopelliti et al. 2024). Baseline characteristics and 6‐month neuropsychiatric outcomes were compared across the three identified clusters using Chi‐square test for categorical variables and Kruskal–Wallis test for quantitative variables. Post‐hoc analyses with Bonferroni correction were applied to continuous variables. To assess the predictive variables associated with each cluster, baseline characteristics that differed between the three clusters with *p *< 0.05 in univariate analyses were included in a multivariable logistic regression model. The absence of collinearity between candidate variables was verified by calculating the variance inflation factors (VIF). Statistical tests were conducted using a two‐tailed significance level of *α* = 0.05.

## 3 Results

### 3.1 Study Cohort

**FIGURE 1:**
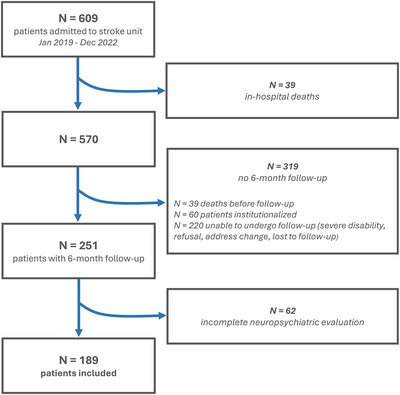
Flowchart of patient inclusion.

A total of 609 patients were consecutively admitted to our Stroke Unit for an acute cerebrovascular event during the study period, 251 of whom were alive and able to receive neuropsychiatric follow‐up 6 months after the index event (Figure 1). Sixty‐two patients did not receive a complete neuropsychiatric evaluation and were excluded from the study. Tables S2 and S3 compare baseline characteristics between patients included and excluded from the study.

A total of 189 patients were finally included in the analysis. Median age of participants was 75.4 years (IQR 67.0–82.3), 118 (62.4%) were male; for 152 (80.4%) an ischemic stroke was the index cerebrovascular event. CT scans were obtained for all patients, while MRI was performed on 137 patients (76%).

### 3.2 Depressive Symptoms Profiles

**FIGURE 2:**
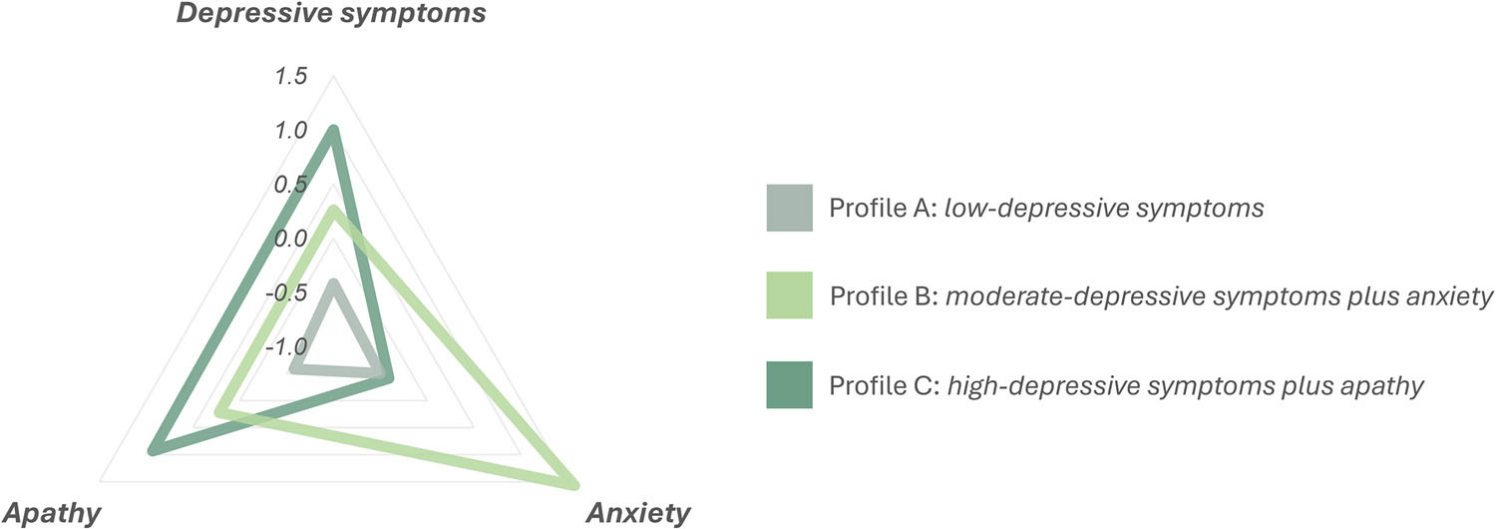
Graphic depiction of depressive symptoms profiles. The radar shows the *z*‐scores of CES‐D scale, NPI‐Q apathy, and NPI‐Q anxiety scores of each cluster.

Using k‐means cluster analysis, we identified three depressive symptoms profiles (Table S4): Profile A, low‐depressive symptoms (*n* = 108 patients, 57.1%); Profile B, moderate‐depressive symptoms plus anxiety (*n* = 41 patients, 21.7%); Profile C, high‐depressive symptoms plus apathy (*n* = 40 patients, 21.2%). Figure 2 shows a graphic depiction of the three neuropsychiatric clusters.

### 3.3 Baseline Characteristics

Table 1 shows the baseline characteristics of patients according to their depressive symptoms profile. In univariate analyses the baseline clinical and neuroimaging variables that were significantly different between the three profiles were the prevalence of pre‐stroke depressive symptoms (*p* = 0.021), years of formal education (*p* = 0.024), global cerebral atrophy score (*p* = 0.009), and both raw (*p* = 0.011) and adjusted (*p* = 0.034) MoCA scores.

**TABLE 1:** Patient characteristics according to depressive symptoms profile.

	Profile A (low depressive symptoms) *N* = 108	Profile B (moderate depressive symptoms + anxiety) *N* = 41	Profile C (high depressive symptoms + apathy) *N* = 40	*p* value
**Baseline characteristics**				
Age, median (IQR)	75.8 (65.2–82.6)	72.8 (64.3–80.1)	77.0 (70.5–82.9)	0.191
Male sex, *n* (%)	73 (67.6)	22 (53.7)	23 (57.5)	0.225
Pre‐stroke depressive symptoms, *n* (%) [*n* = 180]	22 (21.0)	13 (34.2)	16 (43.2)	0.021^ac^
Pre‐stroke disability, *n* (%) [*n* = 188]	5 (4.7)	1 (2.4)	5 (12.5)	0.114
Pre‐stroke IQCODE, median (IQR) [*n* = 157]	3.0 (3.0–3.3)	3.1 (3.0–3.3)	3.0 (3.0–3.4)	0.871
Years of education, median (IQR) [*n* = 187]	11.0 (8.0–13.0)	8.0 (6.5–13.0)	8.0 (5.0–11.0)	0.024^ac^
Hypertension, *n* (%) [*n* = 188]	79 (73.1)	24 (60.0)	31 (77.5)	0.180
Diabetes, *n* (%) [*n* = 188]	26 (24.1)	4 (10.0)	13 (32.5)	0.051
Hypercholesterolemia, *n* (%) [*n* = 188]	72 (66.7)	25 (62.5)	26 (65.0)	0.892
Atrial fibrillation, *n* (%) [*n* = 188]	14 (13.0)	8 (20.0)	4 (10.0)	0.399
**Acute stroke characteristics**				
Baseline NIHSS score, median (IQR)	2.0 (1.0–4.0)	2.0 (0.5–4.5)	2.0 (0.0–5.0)	0.900
Ischemic stroke, *n* (%)	86 (79.6)	34 (82.9)	32 (80.0)	0.900
Transient ischemic attack, *n* (%)	14 (13.0)	4 (9.8)	4 (10.0)	0.807
Intracerebral hemorrhage, *n* (%)	5 (4.6)	2 (4.9)	4 (10.0)	0.445
Other stroke type, *n* (%)	3 (2.8)	1 (2.4)	0 (0.0)	NA
Left hemisphere lesion, *n* (%)	42 (38.9)	18 (43.9)	24 (60.0)	0.072
Raw MoCA score, median (IQR) [*n* = 138]	21.5 (16.0–24.0)	21.0 (16.0–23.0)	16.0 (10.0–21.3)	0.011^ac^
Adjusted MoCA score, median (IQR) [*n* = 136]	22.0 (18.8–24.7)	21.5 (19.2–23.4)	18.1 (13.2–22.9)	0.034^ac^
**Neuroimaging features**				
Van Swieten scale, median (IQR) [*n* = 188]	2.0 (1.0–3.0)	1.0 (0.0–2.0)	2.0 (1.0–3.0)	0.133
Global cerebral atrophy score, median (IQR) [*n* = 188]	10.0 (3.0–17.0)	7.0 (2.0–13.0)	12.0 (8.3–17.0)	0.009^bc^
Cerebral microbleeds ≥1, *n* (%) [*n* = 130]	27 (38.0)	12 (38.7)	8 (28.6)	0.640
Strictly deep*, n* (%)	9 (33.3)	4 (33.3)	1 (12.5)	0.502
Strictly lobar*, n* (%)	12 (44.4)	5 (41.7)	3 (37.5)	0.939
Mixed, *n* (%)	6 (22.2)	3 (25.0)	4 (50.0)	0.296
Cortical superficial siderosis, *n* (%) [*n* = 130]	2 (2.8)	1 (3.2)	0 (0.0)	NA
Fazekas score, median (IQR) [*n* = 137]	2.0 (1.0–3.0)	2.0 (1.0–2.5)	2.0 (1.0–3.0)	0.340

*Note*: The p‐value was calculated using Chi‐square test for categorical variables and Kruskall–Wallis test for continuous variables. One‐to‐one comparisons (Bonferroni correction was used for continuous variables): ab = *p *< 0.05 for comparison Profile A vs. Profile B; ac = *p* < 0.05 for comparison Profile A vs. Profile C; bc = *p* < 0.05 for comparison Profile B vs. Profile C.

Abbreviation: IQCODE = Informant Questionnaire on Cognitive Decline in the Elderly, IQR = interquartile range, MoCA = Montreal Cognitive Assessment, NIHSS = National Institutes of Health Stroke Scale.

In a multivariate logistic regression model (Table 2), compared to patients with low‐depressive symptoms, patients with high‐depressive symptoms plus apathy exhibited lower MoCA scores at baseline (adjusted odds ratio [adj.OR] 0.91, 95% confidence interval [95% CI] 0.83–0.99). Patients with moderate‐depressive symptoms plus anxiety had lower GCA scores compared to both low‐depressive symptoms (adj.OR 0.92, 95% CI 0.86–0.99) and high‐depressive symptoms plus apathy (adj.OR 0.89, 95% CI 0.81–0.97) profiles.

**TABLE 2:** Multivariable model—predictors.

	Profile B (moderate‐depressive symptoms + anxiety) versus Profile A (low‐depressive symptoms)	Profile C (high‐depressive symptoms + apathy) versus Profile A (low‐depressive symptoms)	Profile C (high‐depressive symptoms + apathy) versus Profile B (moderate‐depressive symptoms + anxiety)
Education (per 1‐year increase)	0.91 (0.80–1.03)	0.96 (0.85–1.08)	1.00 (0.86–1.16)
Pre‐stroke depressive symptoms	2.5 (0.93–7.00)	1.98 (0.69–5.71)	0.49 (0.12–1.96)
Baseline raw MoCA score (per 1–point increase)	0.97 (0.88–1.07)	**0.91 (0.83–0.99)**	0.94 (0.84–1.05)
Global cortical atrophy score (per 1‐point increase)	**0.92 (0.86–0.99)**	1.03 (0.97–1.09)	**1.13 (1.03–1.23)**

*Note*: Results of logistic regression analysis including the baseline variables associated with *p *> 0.05 at univariate analysis as covariates. Effect sizes were shown as odds ratio (95% confidence interval). Statistically significant results with *p* value < 0.05 are shown in bold.

### 3.4 Cognitive Outcomes

Table 3 shows the cognitive outcomes of patients at 6‐month follow‐up according to their depressive symptoms profile group. Compared to patients with low depressive symptoms, patients with high‐depressive symptoms plus apathy—but not patients with moderate‐depressive symptoms plus anxiety—showed higher rates of PSCI (OR 2.98, 95% CI 1.14–7.77). Patients with depressive symptoms plus apathy had higher rates of dementia compared to both patients with low‐depressive symptoms (*p* < 0.001) and with moderate‐depressive symptoms plus anxiety (*p* = 0.002).

**TABLE 3:** Cognitive and functional outcomes according to depressive symptoms profile.

6‐month outcomes	A Low‐depressive symptoms	B Moderate‐depressive symptoms + anxiety	C High‐depressive symptoms + apathy	*p* value
Post‐stroke cognitive impairment, *n* (%)	68 (64.2)	27 (69.2)	32 (84.2)	0.071^ac^
Post‐stroke MCI, *n* (%)	48 (45.3)	18 (46.2)	10 (26.3)	0.101^ac^
Post‐stroke dementia, *n* (%)	20 (18.9)	9 (23.1)	22 (57.9)	< 0.001^ac, bc^
Modified Rankin Scale, median (IQR)	1 (0–2)	1 (1–2)	2 (1–3)	0.001^ac, bc^
Disability, *n* (%)	34 (31.5)	13 (31.7)	24 (60.0)	0.004^ac, bc^
Cognitive domains				
Impaired memory, *n* (%)	30 (28.3)	18 (43.9)	16 (41.0)	0.126
Impaired visuospatial ability, *n* (%)	35 (34.3)	10 (30.3)	16 (47.1)	0.302
Impaired language*, n* (%)	21 (19.6)	12 (29.3)	14 (36.8)	0.089^ac^
Impaired attention/executive function, *n* (%)	56 (54.4)	19 (51.4)	28 (75.7)	0.050^ac, bc^

*Note*: The *p* values were calculated using Chi‐square test for categorical variables and Kruskall–Wallis test for continuous variables. One‐to‐one comparisons (Bonferroni correction was used for continuous variables): ab = *p* < 0.05 for Profile A versus Profile B; ac = *p* < 0.05 for Profile A versus Profile C; bc = *p* < 0.05 for Profile B versus. Profile C. Disability at 6‐month follow‐up was defined as a Modified Rankin Scale score > 2.

Abbreviation: IQR = interquartile range.

Patients with high‐depressive symptoms plus apathy had higher median mRS scores and more disability at 6‐month follow‐up compared to the other two groups (all *p* < 0.005).

**FIGURE 3:**
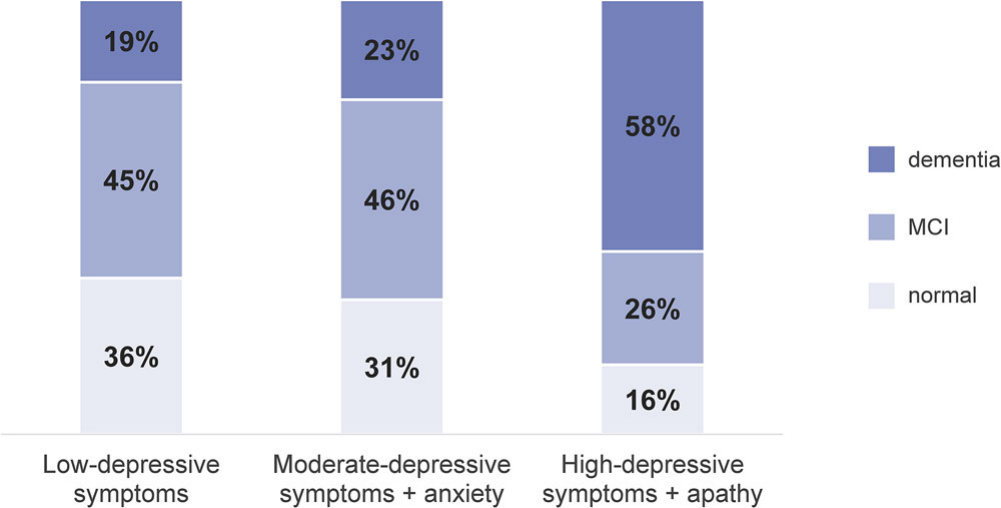
Degree of cognitive impairment across the three depressive symptoms profiles. MCI = mild cognitive impairment.

**FIGURE 4:**
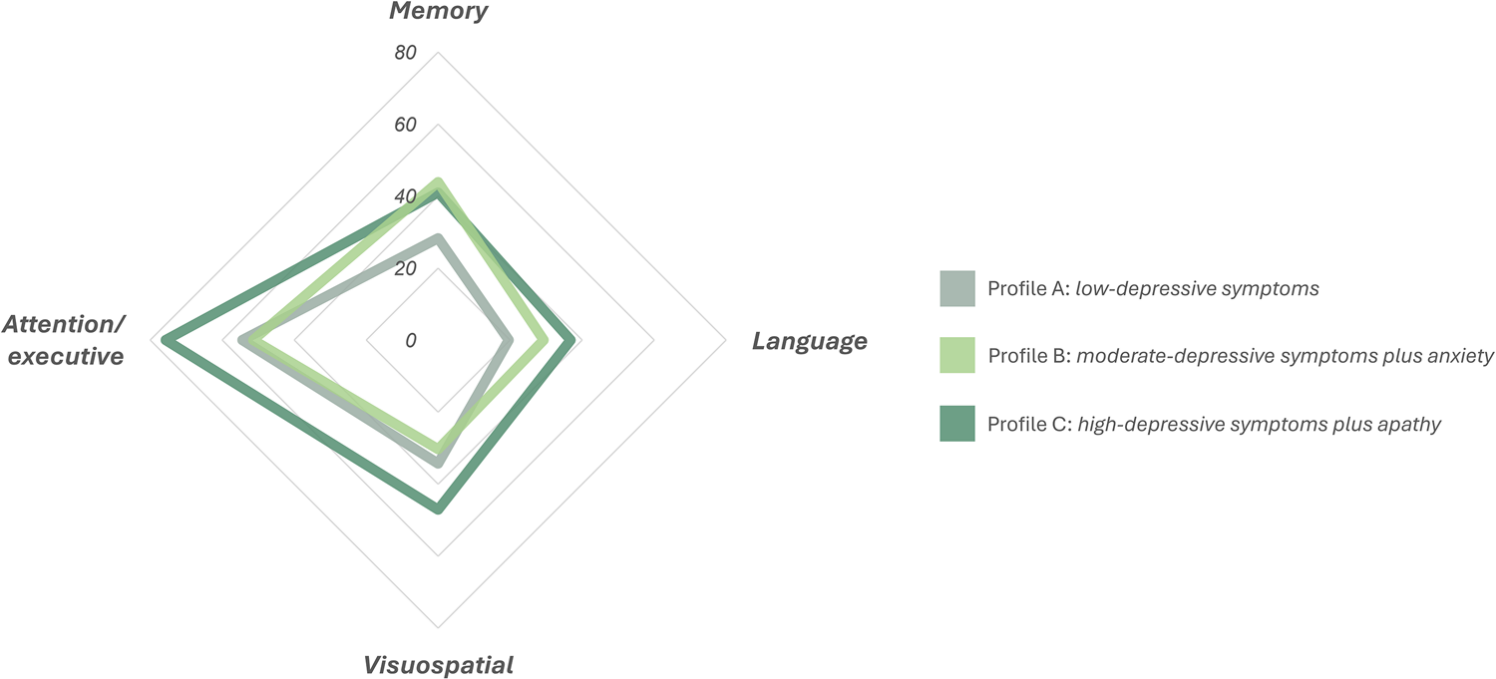
Affected cognitive domains across the three depressive symptoms profiles. The radar depicts the percentage of subjects in each subgroup showing impairment in specific cognitive domains.

Concerning cognitive domains, patients with depressive symptoms plus apathy had higher rates of language impairment compared to patients with low‐depressive symptoms (*p* = 0.033), and higher rates of impaired attentive/executive functions compared to both patients with low‐depressive symptoms (*p* = 0.023) and moderate‐depressive symptoms plus anxiety (*p* = 0.030). Figure 3 shows the rates of MCI and dementia at 6‐month follow‐up, while Figure 4 shows the cognitive domains differently affected across the three groups.

## 4 Discussion

The three depressive symptom profiles that we identified in stroke survivors 6 months after the index event using cluster analysis showed different baseline clinical and neuroimaging characteristics. While patients with moderate depressive symptoms and anxiety exhibited levels of PSCI similar to those in the low‐depressive symptoms group, patients with high depressive symptoms and apathy had the worst overall cognitive performance, with a distinct pattern of domain‐specific impairment. Our results confirm that post‐stroke depression is a heterogeneous condition with distinct subtypes.

Depressive symptoms are among the most common complaints following a stroke and have been closely linked to cognitive decline (Towfighi et al. 2017; Kanellopoulos et al. 2020). A notable heterogeneity in the presentation of post‐stroke depression has been observed, with apathy and anxiety often co‐occurring (Ayerbe et al. 2013; Tay et al. 2020). We hypothesized that apathetic and anxious features could help better characterize post‐stroke depressive symptoms, potentially identifying the neuropsychiatric syndromes most closely associated with PSCI. In line with a previous study on depressive symptoms following intracerebral hemorrhage (Scopelliti et al. 2024), our cohort—consisting primarily of ischemic stroke cases—revealed three distinct depressive symptom profiles, identified based on a cluster analysis incorporating the levels of apathy and anxiety assessed through simple neuropsychiatric questionnaires administered 6 months after the index event.

The profile with high‐depressive symptoms and apathy was linked to poorer 6‐month functional outcomes and cognitive performances, particularly in the domain of attention and executive functions. These patients demonstrated worse cognitive performance already in the acute stroke phase (evaluated using a simple tool such as the MoCA), suggesting the potential for early identification of post‐stroke neuropsychiatric profiles. These findings align with previous research identifying apathy, rather than other mood disorders, as a key factor associated with cognitive decline: apathy and cognitive decline may both reflect underlying damage to the frontal‐subcortical circuits, which are critical for goal‐directed behavior and executive control (Tay et al. 2021; Lohner et al. 2017). Moreover, apathetic symptoms are common features of late‐life depression, a clinical entity exhibiting a lower response to pharmacological treatments, and carrying a high risk for cognitive decline (Steffens et al. 2022; Alexopoulos et al. 2013). From this perspective, apathetic symptoms in elderly stroke patients should be considered as a distinct clinical entity from mood disorders (Scopelliti et al. 2022a; Tay et al. 2020; Alexopoulos et al. 2013). In our study, the cognitive profile of patients with high‐depressive symptoms plus apathy—preferentially affecting attention and executive functions—might be indicative of an underlying vascular cause rather than neurodegeneration, suggesting a link between late‐life depression, vascular cognitive impairment, and distinctive neuropsychiatric manifestations (Alexopoulos 2019). Further studies employing advanced neuroimaging techniques and biological markers of neurodegeneration may help clarifying the neurobiological substrate of the different post‐stroke neuropsychiatric syndromes.

In contrast, the moderate‐depressive symptoms plus anxiety and the low‐depressive symptoms profiles exhibited similar levels of cognitive decline. Moreover, patients with moderate‐depressive symptoms plus anxiety demonstrated lower levels of global cortical atrophy. While anxiety is commonly reported as one of the most frequent neuropsychiatric manifestations following a stroke and often co‐morbid with depression, it might be associated more with emotional distress and coping mechanisms related to stroke symptoms, rather than cognitive impairment (Kapoor et al. 2019; Chun et al. 2018; Scopelliti et al. 2022b). Despite neuropsychiatric and cognitive disturbances identified after stroke are complex and multifaced, possibly influenced by both acute stroke‐related and chronic brain changes, our findings suggest that depressive symptoms with prevalent anxiety are less likely to serve as markers of vascular cognitive impairment compared to those with more pronounced apathy.

The strength of our study lies in the comprehensive assessment of patients' cognitive status, which included an extensive neuropsychological battery administered 6 months after the index stroke. However, there are some limitations to consider. The sample size was relatively small, potentially limiting the generalizability of our findings. In addition, the cross‐sectional design of the neuropsychiatric evaluation constrains our ability to determine the long‐term trajectories of depressive symptoms and cognitive decline. Future longitudinal studies are needed to investigate how these depressive symptom profiles evolve over time and their long‐term impact on cognition. We did not assess relevant imaging markers of small vessel disease, such as lacunes and enlarged perivascular spaces, nor did we collect detailed information on lesion location beyond the hemisphere affected. In addition, data on prior history of stroke were not available. These factors might influence baseline cognitive status and post‐stroke neuropsychiatric outcomes, and should be addressed in future studies. Also, we did not collect reliable information on prior psychiatric diagnoses or on the use of psychotropic treatments before or after the index stroke, which may have influenced both baseline and follow‐up neuropsychiatric assessments. We acknowledge that our findings may only apply to a subset of less compromised patients, as we excluded a substantial proportion of patients who did not undergo the 6‐month neuropsychiatric screening: these excluded patients were older, had more comorbidities, higher levels of pre‐stroke cognitive impairment, and more severe index strokes. Moreover, the majority of included patients had ischemic strokes, which may limit the generalizability of our findings to other types of cerebrovascular events, such as hemorrhagic strokes or TIA. Furthermore, the neuropsychiatric profiles were derived from cluster analyses based on screening tools, rather than comprehensive clinical evaluations, making it difficult to establish formal clinical diagnoses and depict the multidimensional nature of neuropsychiatric symptoms. However, the CES‐D scale and NPI‐Q are simple tools that can be easily administered in clinical settings, offering a quick and reliable means of evaluating neuropsychiatric symptoms in stroke patients. Validating these clusters in independent cohorts and applying alternative clustering approaches—e.g., hierarchical clustering—could help strengthening the robustness and generalizability of our findings.

In conclusion, the differentiation of depressive symptoms profiles—by including apathy and anxiety features in a cluster analysis—allowed us to uncover nuances in post‐stroke neuropsychiatric outcomes that are often missed in commonly used screening tests. These findings highlight how different neuropsychiatric syndromes after stroke may recognize distinct underlying mechanisms and cognitive outcomes, reinforcing the value of comprehensive, multidimensional assessments to better inform clinical management and care strategies. Future studies with extended follow‐up will be essential to determine the trajectories of depressive symptom profiles over time, and to clarify their long‐term prognostic implications.

## Author Contributions

**Giuseppe Scopelliti**: conceptualization, investigation, writing–original draft, methodology, visualization, formal analysis, validation, data curation. **Francesco Mele**: investigation, writing–review and editing, data curation, conceptualization. **Ilaria Cova**: conceptualization, writing–review and editing, data curation. **Federico Masserini**: investigation, writing–review and editing, data curation. **Valentina Cucumo**: writing–review and editing, data curation. **Giorgia Maestri**: writing–review and editing, data curation. **Alessia Nicotra**: writing–review and editing, data curation. **Arianna Forgione**: writing–review and editing, data curation. **Pierluigi Bertora**: writing–review and editing. **Simone Pomati**: writing–review and editing, data curation. **Emilia Salvadori**: conceptualization, writing–review and editing, data curation, supervision, methodology, formal analysis, investigation. **Leonardo Pantoni**: conceptualization, writing–review and editing, project administration, resources, supervision.

## Ethics Statement

All patients provided informed consent to participate in any assessments required for clinical practice, including neuroimaging. The local institutional review board later approved the retrospective analysis of the data. All procedures were conducted in accordance with the Declaration of Helsinki. The corresponding author had full access to the study's data and is responsible for its integrity and analysis. The data supporting the findings of this study are available from the corresponding author upon reasonable request. This article adheres to the guidelines of the Strengthening the Reporting of Observational Studies in Epidemiology.

## Conflicts of Interest

Leonardo Pantoni has received consultation fees from Amicus, Medtronic, and PIAM. The other authors declare no conflicts of interest.

## Peer Review

The peer review history for this article is available at https://publons.com/publon/10.1002/brb3.70801.

## Supporting information




**Supplemental Materials**: brb370801‐sup‐0001‐SuppMat.docx

## Data Availability

The data that support the findings of this study are available on request from the corresponding author. The data are not publicly available due to privacy or ethical restrictions.

## References

[brb370801-bib-0001] Aiello, E. N. , C. Gramegna , A. Esposito , et al. 2022. “The Montreal Cognitive Assessment (MoCA): Updated Norms and Psychometric Insights Into Adaptive Testing From Healthy Individuals in Northern Italy.” Aging Clinical and Experimental Research 34, no. 2: 375–382. 10.1007/S40520-021-01943-7. 34313961 PMC8847194

[brb370801-bib-0002] Alexopoulos, G. S. 2019. “Mechanisms and Treatment of Late‐Life Depression.” Translational Psychiatry 9, no. 1: 188. 10.1038/S41398-019-0514-6. 31383842 PMC6683149

[brb370801-bib-0003] Alexopoulos, G. S. , M. J. Hoptman , G. Yuen , et al. 2013. “Functional Connectivity in Apathy of Late‐Life Depression: A Preliminary Study.” Journal of Affective Disorders 149, no. 1–3: 398–405. 10.1016/J.JAD.2012.11.023. 23261142 PMC3636174

[brb370801-bib-0004] Ayerbe, L. , S. Ayis , C. D. A. Wolfe , and A. G. Rudd . 2013. “Natural History, Predictors and Outcomes of Depression After Stroke: Systematic Review and Meta‐Analysis.” British Journal of Psychiatry 202, no. 1: 14–21. 10.1192/BJP.BP.111.107664. 23284148

[brb370801-bib-0005] Ayerbe, L. , S. A. Ayis , S. Crichton , C. D. A. Wolfe , and A. G. Rudd . 2014. “Natural History, Predictors and Associated Outcomes of Anxiety up to 10 Years After Stroke: The South London Stroke Register.” Age and Ageing 43, no. 4: 542–547. 10.1093/ageing/aft208. 24375225

[brb370801-bib-0006] Capitani, E. , and M. Laiacona . 1997. “Composite Neuropsychological Batteries and Demographic Correction: Standardization Based on Equivalent Scores, With a Review of Published Data. The Italian Group for the Neuropsychological Study of Ageing.” Journal of Clinical and Experimental Neuropsychology 19, no. 6: 795–809. 10.1080/01688639708403761. 9524875

[brb370801-bib-0007] Castello, J. P. , M. Pasi , P. Kubiszewski , et al. 2022. “Cerebral Small Vessel Disease and Depression Among Intracerebral Hemorrhage Survivors.” Stroke 53, no. 2: 523–531. 10.1161/STROKEAHA.121.035488. 34587793 PMC8792169

[brb370801-bib-0008] Chun, H. Y. Y. , W. N. Whiteley , M. S. Dennis , G. E. Mead , and A. J. Carson . 2018. “Anxiety After Stroke the Importance of Subtyping.” Stroke 49, no. 3: 556–564. 10.1161/STROKEAHA.117.020078/-/DC1. 29437982 PMC5839706

[brb370801-bib-0009] Cova, I. , F. Mele , A. Nicotra , et al. 2024. “The Luigi Sacco Hospital VAS‐COG Stroke Care Pathway: A Five‐Year Experience.” Cerebral Circulation ‐ Cognition and Behavior 6: 100210. 10.1016/J.CCCB.2024.100210. 38357360 PMC10865214

[brb370801-bib-0010] Cova, I. , F. Mele , F. Zerini , et al. 2022. “The Clock Drawing Test as a Predictor of Cognitive Decline in Non‐Demented Stroke Patients.” Journal of Neurology 269, no. 1: 342–349. 10.1007/S00415-021-10637-Z. 34095964 PMC8739305

[brb370801-bib-0011] Cummings, J. L. , M. Mega , K. Gray , S. Rosenberg‐Thompson , D. A. Carusi , and J. Gornbein . 1994. “The Neuropsychiatric Inventory: Comprehensive Assessment of Psychopathology in Dementia.” Neurology 44, no. 12: 2308–2314. 10.1212/wnl.44.12.2308. 7991117

[brb370801-bib-0012] Fazekas, F. , J. B. Chawluk , A. Alavi , H. I. Hurtig , and R. A. Zimmerman . 1987. “MR Signal Abnormalities at 1.5 T in Alzheimer's Dementia and Normal Aging.” American Journal of Roentgenology 149, no. 2: 351–356. 10.2214/ajr.149.2.351. 3496763

[brb370801-bib-0013] Hobden, G. , E. Colbourne , S. T. Pendlebury , and N. Demeyere . 2024. “Reliability of the Global Cortical Atrophy Visual Rating Scale Applied to Computed Tomography Versus Magnetic Resonance Imaging Scans in Acute Stroke.” Neurological Sciences 45, no. 4: 1549–1556. 10.1007/S10072-023-07113-Z. 37910322 PMC10942897

[brb370801-bib-0014] Jorm, A. F. 1994. “A Short Form of the Informant Questionnaire on Cognitive Decline in the Elderly (IQCODE): Development and Cross‐Validation.” Psychological Medicine 24, no. 1: 145–153. 10.1017/S003329170002691X. 8208879

[brb370801-bib-0015] Kanellopoulos, D. , V. Wilkins , J. Avari , et al. 2020. “Dimensions of Post‐Stroke Depression and Neuropsychological Deficits in Older Adults HHS Public Access.” American Journal of Geriatric Psychiatry 28, no. 7: 764–771. 10.1016/j.jagp.2020.01.009. PMC7354891 32081532

[brb370801-bib-0016] Kapoor, A. , K. Si , A. Y. X. Yu , et al. 2019. “Younger Age and Depressive Symptoms Predict High Risk of Generalized Anxiety After Stroke and Transient Ischemic Attack.” Stroke 50, no. 9: 2359–2363. 10.1161/STROKEAHA.119.025464. 31405330

[brb370801-bib-0017] Kutlubaev, M. A. , and M. L. Hackett . 2014. “Part II: Predictors of Depression After Stroke and Impact of Depression on Stroke Outcome: An Updated Systematic Review of Observational Studies.” International Journal of Stroke 9, no. 8: 1026–1036. 10.1111/IJS.12356. 25156411

[brb370801-bib-0018] Lohner, V. , R. L. Brookes , M. J. Hollocks , R. G. Morris , and H. S. Markus . 2017. “Apathy, but Not Depression, Is Associated With Executive Dysfunction in Cerebral Small Vessel Disease.” PLoS ONE 12, no. 5: e0176943. 10.1371/JOURNAL.PONE.0176943. 28493898 PMC5426624

[brb370801-bib-0019] Mele, F. , I. Cova , A. Nicotra , et al. 2024. “Prestroke Cognitive Impairment: Frequency and Association With Premorbid Neuropsychiatric, Functional, and Neuroimaging Features.” Stroke 55: 1869–1876. 10.1161/STROKEAHA.123.045344/SUPPL_FILE/STR_STROKE-2023-045344_SUPP2.PDF. 38818731 PMC11198949

[brb370801-bib-0020] Nasreddine, Z. S. , N. A. Phillips , V. Bédirian , et al. 2005. “The Montreal Cognitive Assessment, MoCA: A Brief Screening Tool for Mild Cognitive Impairment.” Journal of the American Geriatrics Society 53, no. 4: 695–699. 10.1111/J.1532-5415.2005.53221.X. 15817019

[brb370801-bib-0021] Pasquier, F. , D. Leys , J. G. E. Weerts , F. Mounier‐Vehier , F. Barkhof , and P. Scheltens . 1996. “Inter‐ and Intraobserver Reproducibility of Cerebral Atrophy Assessment on MRI Scans With Hemispheric Infarcts.” European Neurology 36, no. 5: 268–272. 10.1159/000117270. 8864706

[brb370801-bib-0022] Scopelliti, G. , B. Casolla , G. Boulouis , et al. 2022b. “Long‐Term Anxiety in Spontaneous Intracerebral Hemorrhage Survivors.” International Journal of Stroke 17, no. 10: 1093–1099. 10.1177/17474930221085443/SUPPL_FILE/SJ-DOCX-1-WSO-10.1177_17474930221085443.DOCX. 35187993

[brb370801-bib-0023] Scopelliti, G. , B. Casolla , G. Boulouis , et al. 2022a. “Long‐Term Neuropsychiatric Symptoms in Spontaneous Intracerebral Haemorrhage Survivors.” Journal of Neurology, Neurosurgery, and Psychiatry 93, no. 3: 232–237. 10.1136/JNNP-2021-327557. 34728587

[brb370801-bib-0024] Scopelliti, G. , M. Kyheng , B. Casolla , et al. 2024. “Depressive Symptoms Profile and Dementia Risk After Spontaneous Intracerebral Haemorrhage.” European Stroke Journal 10: 610–617. 10.1177/23969873241284725. 39324780 PMC11556666

[brb370801-bib-0025] Shinar, D. , C. R. Gross , T. R. Price , M. Banko , P. L. Bolduc , and R. G. Robinson . 1986. “Screening for Depression in Stroke Patients: The Reliability and Validity of the Center for Epidemiologic Studies Depression Scale.” Stroke 17, no. 2: 241–245. 10.1161/01.STR.17.2.241. 3961834

[brb370801-bib-0026] Steffens, D. C. , M. Fahed , K. J. Manning , and L. Wang . 2022. “The Neurobiology of Apathy in Depression and Neurocognitive Impairment in Older Adults: A Review of Epidemiological, Clinical, Neuropsychological and Biological Research.” Translational Psychiatry 12, no. 1: 1–16. 10.1038/s41398-022-02292-3. 36572691 PMC9792580

[brb370801-bib-0027] Tay, J. , R. G. Morris , and H. S. Markus . 2021. “Apathy After Stroke: Diagnosis, Mechanisms, Consequences, and Treatment.” International Journal of Stroke 16, no. 5: 510–518. https://pubmed.ncbi.nlm.nih.gov/33527880/. 33527880 10.1177/1747493021990906 PMC8267086

[brb370801-bib-0028] Tay, J. , R. G. Morris , A. M. Tuladhar , M. Husain , F. E. De Leeuw , and H. S. Markus . 2020. “Apathy, but Not Depression, Predicts All‐Cause Dementia in Cerebral Small Vessel Disease.” Journal of Neurology, Neurosurgery, and Psychiatry 91, no. 9: 953–959. 10.1136/jnnp-2020-323092. 32651249 PMC7476304

[brb370801-bib-0029] Towfighi, A. , B. Ovbiagele , and N. El Husseini , et al. 2017. “Poststroke Depression: A Scientific Statement for Healthcare Professionals From the American Heart Association/American Stroke Association.” Stroke 48, no. 2: e30–e43. 10.1161/STR.0000000000000113. 27932603

[brb370801-bib-0030] Van Swieten, J. C. , J. Van Gijn , A. Hijdra , and P. J. Koudstaal . 1990. “Grading White Matter Lesions on CT and MRI: A Simple Scale.” Journal of Neurology, Neurosurgery, and Psychiatry 53, no. 12: 1080–1083. 10.1136/JNNP.53.12.1080. 2292703 PMC488320

[brb370801-bib-0031] Wardlaw, J. M. , E. E. Smith , G. J. Biessels , et al. 2013. “Neuroimaging Standards for Research Into Small Vessel Disease and Its Contribution to Ageing and Neurodegeneration.” Lancet Neurology 12, no. 8: 822–838. 10.1016/S1474-4422(13)70124-8. 23867200 PMC3714437

[brb370801-bib-0032] Xiao, W. , Y. Liu , J. Huang , L. A. Huang , Y. Bian , and G. Zou . 2024. “Analysis of Factors Associated With Depressive Symptoms in Stroke Patients Based on a National Cross‐Sectional Study.” Scientific Reports 14, no. 1: 9268. 10.1038/S41598-024-59837-3. 38649386 PMC11035548

[brb370801-bib-0033] Zhang, S. , M. Xu , Z. J. Liu , J. Feng , and Y. Ma . 2020. “Neuropsychiatric Issues After Stroke: Clinical Significance and Therapeutic Implications.” World Journal of Psychiatry 10, no. 6: 125–138. 10.5498/wjp.v10.i6.125. 32742946 PMC7360525

