# Supplementary material for: Depressive Symptoms Profiles and Cognitive Outcomes After Stroke
Source: Brain Behav. 2025 Sep 15;15(9):e70801. doi: 10.1002/brb3.70801 (PMC12434606; doi:10.1002/brb3.70801)
Supplement: Supplementary file 1 — Supplemental Materials: brb370801‐sup‐0001‐SuppMat.docx [file BRB3-15-e70801-s001.docx]

***SUPPLEMENTAL MATERIALS***

**Depressive symptoms profiles and cognitive outcomes after stroke**

Giuseppe Scopelliti,^1^ Francesco Mele,^1^ Ilaria Cova,^1^ Federico Masserini,^1^ Valentina Cucumo,^1^ Giorgia Maestri,^1^ Alessia Nicotra,^1^ Arianna Forgione,^1^ Pierluigi Bertora,^2^ Simone Pomati,^1^ Emilia Salvadori,^2^ Leonardo Pantoni^2,3^

1. Neurology Unit, Luigi Sacco University Hospital, Milan, Italy.

2. Neuroscience Research Center, Department of Biomedical and Clinical Sciences, University of Milan, Milan, Italy.

3. Department of Neurorehabilitation, Casa di Cura Igea, Milan, Italy

*Corresponding author:* Prof. Leonardo Pantoni

Address: Department of Biomedical and Clinical Sciences, University of Milan, Milan, Italy Email: [leonardo.pantoni@unimi.it](mailto:leonardo.pantoni@unimi.it)

***Contents:*** *Supplemental tables 1, 2, 3*

***Supplemental table 1.*** **Neuropsychological tests administered at 6-month follow-up**

| **Cognitive domains** | **Neuropsychological tests** |
| --- | --- |
| *MEMORY* | 1. *Rey auditory verbal learning test (immediate recall)* 2. *Rey auditory verbal learning test (delayed recall)* 3. *Rey-Osterrieth complex figure (delayed recall)* |
| *VISUOSPATIAL ABILITY* | 1. *Rey-Osterrieth complex figure (copy)* |
| *LANGUAGE* | 1. *Phonemic verbal fluency test* 2. *Semantic verbal fluency test* 3. *Phrase construction test* |
| *ATTENTION/EXECUTIVE FUNCTION* | 1. *Trail Making Test A* 2. *Trail Making Test B* 3. *Stroop test* |

***Supplemental table 2.* Characteristics of patients included vs. all patients excluded**

|  | **Included**  **N = 189** | **Excluded**  **N = 420** | ***p-value*** |
| --- | --- | --- | --- |
| ***Patient characteristics*** |  |  |  |
| Age, *median (IQR)* | 75.4 (67.0-82.3) | 80.1 (69.7-87.0) | <0.001 |
| Male sex, *n (%)* | 118 (62.4) | 216 (51.4) | 0.012 |
| Pre-stroke disability, *n (%)* | 11 (5.9) | 112 (26.9) | <0.001 |
| Pre-stroke IQCODE, *median (IQR)* | 3.0 (3.0-3.3) | 3.1 (3.0-3.8) | 0.007 |
| Years of education, *median (IQR)* | 8.0 (7.0-13.0) | 8.0 (5.0-13.0) | 0.133 |
| Hypertension, *n (%)* | 134 (71.3) | 320 (76.6) | 0.166 |
| Diabetes, *n (%)* | 43 (22.9) | 118 (28.3) | 0.162 |
| Hypercholesterolemia, *n (%)* | 123 (65.4) | 245 (59.5) | 0.164 |
| Atrial fibrillation, *n (%)* | 26 (13.8) | 104 (24.9) | 0.002 |
| Van Swieten scale, *median (IQR)* | 2.0 (1.0-3.0) | 2.0 (1.0-3.0) | 0.001 |
| Global cerebral atrophy score, *median (IQR)* | 10.0 (4.0-15.0) | 12.0 (6.0-20.0) | 0.001 |
| Ischemic stroke, *n (%)* | 152 (80.4) | 332 (79.0) | 0.697 |
| Transient ischemic attack, *n (%)* | 22 (11.6) | 29 (6.9) | 0.051 |
| Intracerebral hemorrhage, *n (%)* | 11 (5.8) | 50 (11.9) | 0.021 |
| Other stroke type, *n (%)* | 4 (2.1) | 9 (2.1) | 1.000 |
| Baseline NIHSS score, *median (IQR)* | 2.0 (0.0-4.0) | 5.0 (2.0-12.0) | <0.001 |
| Left hemisphere lesion, *n (%)* | 84 (44.4) | 218 (52.2) | 0.079 |
| Raw MOCA score, *median (IQR)* | 19.5 (15.0-24.0) | 17.0 (11.0-22.0) | 0.010 |
| Adjusted MOCA score, *median (IQR)* | 21.6 (17.5-24.3) | 19.3 (14.8-22.8) | 0.003 |

The α value was calculated using chi-square analysis for categorial variables and Mann-Whitney *U* test for continuous variables. IQR = interquartile range. IQCODE = Informant Questionnaire on Cognitive Decline in the Elderly. NIHSS = National Institutes of Health Stroke Scale. MOCA = Montreal Cognitive Assessment. Raw and adjusted MOCA scores were assessed within the first few days after hospitalization for the index stroke.

***Supplemental table 3.* Characteristics of patients included vs. patients excluded for incomplete 6-month neuropsychiatric screening**

|  | **Included**  **N = 189** | **Excluded**  **N = 62** | ***p-value*** |
| --- | --- | --- | --- |
| ***Patient characteristics*** |  |  |  |
| Age, *median (IQR)* | 75.4 (67.0-82.3) | 76.1 (61.8-83.7) | 0.897 |
| Male sex, *n (%)* | 118 (62.4) | 37 (59.7) | 0.698 |
| Pre-stroke disability, *n (%)* | 11 (5.9) | 5 (8.1) | 0.537 |
| Pre-stroke IQCODE, *median (IQR)* | 3.0 (3.0-3.3) | 3.0 (3.0-3.2) | 0.388 |
| Years of education, *median (IQR)* | 8.0 (7.0-13.0) | 8.0 (6.0-13.0) | 0.596 |
| Hypertension, *n (%)* | 134 (71.3) | 48 (77.4) | 0.346 |
| Diabetes, *n (%)* | 43 (22.9) | 13 (21.0) | 0.755 |
| Hypercholesterolemia, *n (%)* | 123 (65.4) | 38 (61.3) | 0.555 |
| Atrial fibrillation, *n (%)* | 26 (13.8) | 9 (14.5) | 0.893 |
| Van Swieten scale, *median (IQR)* | 2.0 (1.0-3.0) | 2.0 (1.0-3.0) | 0.676 |
| Global cerebral atrophy score, *median (IQR)* | 10.0 (4.0-15.0) | 7.0 (4.0-14.0) | 0.277 |
| Ischemic stroke, *n (%)* | 152 (80.4) | 47 (75.8) | 0.436 |
| Transient ischemic attack, *n (%)* | 22 (11.6) | 6 (9.7) | 0.670 |
| Intracerebral hemorrhage, *n (%)* | 11 (5.8) | 5 (8.1) | 0.530 |
| Other stroke type, *n (%)* | 4 (2.1) | 4 (6.5) | 0.092 |
| Baseline NIHSS score, *median (IQR)* | 2.0 (0.0-4.0) | 2.0 (0.0-7.0) | 0.675 |
| Left hemisphere lesion, *n (%)* | 84 (44.4) | 26 (41.9) | 0.730 |
| Raw MOCA score, *median (IQR)* | 19.5 (15.0-24.0) | 20.0 (14.0-24.0) | 0.902 |
| Adjusted MOCA score, *median (IQR)* | 21.6 (17.5-24.3) | 21.6 (17.2-23.9) | 0.842 |

The α value was calculated using chi-square analysis for categorial variables and Mann-Whitney *U* test for continuous variables. IQR = interquartile range. IQCODE = Informant Questionnaire on Cognitive Decline in the Elderly. NIHSS = National Institutes of Health Stroke Scale. MOCA = Montreal Cognitive Assessment. Raw and adjusted MOCA scores were assessed within the first few days after hospitalization for the index stroke.

***Supplemental table 4.* Neuropsychiatric characteristics of the three identified clusters**

|  |  | **I** | | | **II** | | |
| --- | --- | --- | --- | --- | --- | --- | --- |
| **cluster** | **n** | **z_ces-d** | **z_anxiety** | **z_apathy** | **depressed** | **anxious** | **apathetic** |
| **A**  **Low depressive symptoms**  ***N = 108*** | 108 | -0.42 | -0.50 | -0.58 | 29 (27) | 13 (12) | 11 (10) |
| **B**  **Moderate depressive symptoms + anxiety**  **N = 41** | 41 | 0.26 | 1.57 | 0.22 | 23 (56) | 41 (100) | 20 (49) |
| **C**  **High depressive symptoms + apathy**  ***N = 40*** | 40 | 1.00 | -0.41 | 0.93 | 31 (78) | 8 (20) | 35 (88) |

Column-I shows the Z-scores of CES-D depressive symptoms scale, NPI-q apathy, and NPI-q anxiety scores of each neuropsychiatric profiles after clustering; column-II shows the absolute number (percentage) of patients showing significant symptoms according to pre-specified cut-off values (scores ≥16 for CES-D, severity scores ≥1 for NPI-q items)
